# *catena*-Poly[[silver(I)-μ-dipyrazin-2-ylamine] perchlorate monohydrate]

**DOI:** 10.1107/S1600536809037532

**Published:** 2009-10-10

**Authors:** Wei Feng Song, Chong-Qing Wan, Jianfeng Liu

**Affiliations:** a Faculty of Environmental Science and Engineering, Guang Dong University of Technology, Guangzhou 510006, People’s Republic of China; b Deparment of Chemistry, Capital Normal University, Beijing 100048, People’s Republic of China

## Abstract

In the title complex, {[Ag(C_8_H_7_N_5_)]ClO_4_·H_2_O}_*n*_, the multidentate dipyrazin-2-ylamine acts as a μ_2_-bridging link with an *anti*–*syn* configuration, assembling the Ag^I^ ions into a zigzag chain structure. The Ag^I^ ion is linearly coordinated by two dipyrazin-2-ylamine ligands through two pyrazine N atoms. (ClO_4_^−^)⋯π(pyrazine) [O⋯centroid distances of 3.612 (3) and 3.664 (1) Å] and π–π inter­actions [centroid–centroid distance = 3.518 (2) Å] as well as O—H⋯O and N—H⋯O hydrogen-bonds assemble the chains into a three-dimensional supra­molecular aggregation.

## Related literature

For oligo-α-pyridylamino metal-organic frameworks, see: Clérac *et al.* (2000 ▶); Chem *et al.* (2006 ▶). For other dipyrazin-2-ylamine (Hdpza)–metal complexes, see: Ismayilov *et al.* (2007 ▶). For supra­molecular assemblies related to N-rich heterocycles, see: Egli & Sarkhel (2007 ▶); Mooibroek *et al.* (2008 ▶). 
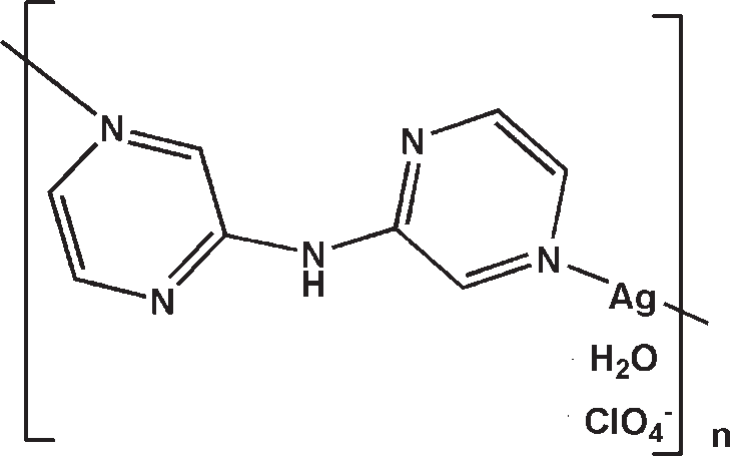


## Experimental

### 

#### Crystal data

[Ag(C_8_H_7_N_5_)]ClO_4_·H_2_O
*M* *_r_* = 398.52
Orthorhombic, 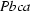

*a* = 9.035 (4) Å
*b* = 15.188 (6) Å
*c* = 18.556 (7) Å
*V* = 2546.4 (17) Å^3^
*Z* = 8
Mo *K*α radiation
μ = 1.82 mm^−1^
*T* = 293 K
0.51 × 0.41 × 0.30 mm

#### Data collection

Bruker SMART CCD area-detector diffractometer
Absorption correction: multi-scan (*SADABS*; Bruker, 1998 ▶) *T*_min_ = 0.36, *T*_max_ = 0.58
16026 measured reflections
3144 independent reflections
1786 reflections with *I* > 2σ(*I*)
*R*_int_ = 0.088

#### Refinement

*R*[*F* ^2^ > 2σ(*F* ^2^)] = 0.068
*wR*(*F* ^2^) = 0.222
*S* = 1.01
3144 reflections
181 parameters
H-atom parameters constrained
Δρ_max_ = 1.68 e Å^−3^
Δρ_min_ = −1.19 e Å^−3^

### 

Data collection: *SMART* (Bruker, 1998 ▶); cell refinement: *SAINT* (Bruker, 1998 ▶); data reduction: *SAINT*; program(s) used to solve structure: *SHELXS97* (Sheldrick, 2008 ▶); program(s) used to refine structure: *SHELXL97* (Sheldrick, 2008 ▶); molecular graphics: *SHELXTL* (Sheldrick, 2008 ▶); software used to prepare material for publication: *SHELXTL* and *PLATON* (Spek, 2009 ▶).

## Supplementary Material

Crystal structure: contains datablocks global, I. DOI: 10.1107/S1600536809037532/bg2275sup1.cif

Structure factors: contains datablocks I. DOI: 10.1107/S1600536809037532/bg2275Isup2.hkl

Additional supplementary materials:  crystallographic information; 3D view; checkCIF report

## Figures and Tables

**Table 1:** Hydrogen-bond geometry (Å, °)

*D*—H⋯*A*	*D*—H	H⋯*A*	*D*⋯*A*	*D*—H⋯*A*
N5—H5⋯O1*W*	0.82	2.12	2.911 (1)	162
O1*W*—H1*WB*⋯O3^i^	0.89	2.21	3.036 (9)	154
O1*W*—H1*WA*⋯O2^ii^	0.89	2.45	3.306 (14)	161
O1*W*—H1*WA*⋯O1^ii^	0.89	2.51	3.063 (11)	121

Symmetry codes: (i) 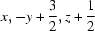; (ii) 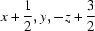.

**Fig. 1.**
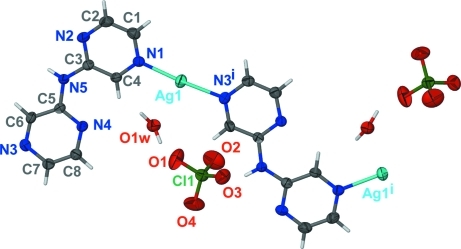
Ellipsoid plot (at the 50% probability level) and atomic numbering scheme of the title complex. [Symmetry code: (i) –x + 3/2, –y + 1, z – 1/2]

**Fig. 2.**
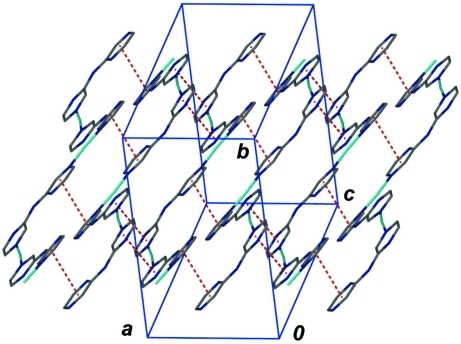
The cationic layer formed by one-dimensional chains linked through π–π interactions. All hydrogen atoms are omitted for clarity. The red dashed lines indicate the π–π interactions.

## supplementary crystallographic information

### Comment

The oligo-α-pyridylamino ligands are widely employed in the construction of diverse interesting metal-organic frameworks (Clérac *et al.*, 2000, Chem *et al.*, 2006). By using one or both nitrogen ligation sites in each heteroaromatic ring attached to the rotatable *C*–*N*(amine) bond, dipyrazin-2-ylamine (Hdpza) has led to several Cu(II), Co(II), Ni(II) and Cr(II) complexes (Ismayilov *et al.*, 2007). Notably, π–acidic aromatic rings such as these N-rich heterocycles have been demonstrated to play an important role in supramolecular assemblies through anion–π interaction, which is of current interest (Egli *et al.*, 2007, Mooibroek *et al.*, 2008).

The asymmetric unit in the title silver(I) complex ([Ag(Hdpza)]^+^.ClO_4_^-^.H_2_O)_∞_ consists of an [Ag(Hdpza)]^+^ cationic group, accompanied by one perchlorate anion and one water solvate (Fig.1). Each Ag^I^ center is surrounded by two Hdpza with two 4-pyrazinyl N atoms [N1 and N3^i^, (i): –*x* + 3/2, -*y* + 1, *z* – 1/2] bonding to the metal, while the ligand exhibits as a µ_2_-bridging mode with the two 4-pyrazinyl N atoms as bonding sites to link the Ag^I^ ions into an infinite chain structure along the *c* axis (Fig.2). Cationic chains are stacked along the *a* axis and interconnect through π–π interactions (Fig. 2). In addition, a O_(perchl)_···π_(pyrazine)_ interaction combines with O-H···O and N-H···O hydrogen-bonds (Table 1) to assemble the infinite chain motifs into a three-dimensional supramolecular structure .

Lattice water molecules and perchlorate anions are embedded within the interstices through *O*—*H*_(water)_···O_(perchl)_ and *N*—*H*_(amine)_···*O*_(water)_ H-bonding. The ClO_4_^-^ anion simultaneously links three neighbouring chains through weak C—H···O_(perchl)_ (C···O span: 3.446 (1) Å - 3.499 (2) Å ) and O_(perchl)_ ···π interactions (O2···Cg: 3.612 (3) Å; O3···Cg: 3.664 (1) Å; Cg:the pyrazinyl ring centroid)

### Experimental

Hdpza was synthesized following literature procedures (Ismayilov *et al.*, 2007). A mixture of Hdpza (100 mg, 0.58 mmol) and AgClO_4_.xH_2_O (172 mg) in methanol (40 ml) was stirred for five hours at room temperature. The resulting clear solution was filtered and then left to stand in air for about 7 days. Brown crystals suitable for X-ray diffraction (97.1 mg, 42% yield, on the basis of Hdpza) were obtained.

### Refinement

Hydrogen atoms attached to C were placed in idealized positions and allowed to ride on the corresponding carbon atoms, with C— H = 0.93 Å and *U*_iso_(H) = 1.2Ueq(C). O-H's and N-H's were obtained from Fourier-difference maps, idealized with a O—H: 0.89 Å , N-H= 0.82 Å and allowed to ride with *U*ĩso~(H) = 1.5Ueq(O).

### Crystal data

**Table:**

[Ag(C_8_H_7_N_5_)]ClO_4_·H_2_O	*F*(000) = 1568
*M**_r_* = 398.52	*D*_x_ = 2.079 Mg m^−^^3^
Orthorhombic, *P**b**c**a*	Mo *K*α radiation, λ = 0.71073 Å
Hall symbol: -P 2ac 2ab	Cell parameters from 834 reflections
*a* = 9.035 (4) Å	θ = 3.0–28.1°
*b* = 15.188 (6) Å	µ = 1.82 mm^−^^1^
*c* = 18.556 (7) Å	*T* = 293 K
*V* = 2546.4 (17) Å^3^	Block, brown
*Z* = 8	0.51 × 0.41 × 0.30 mm

### Data collection

**Table:**

Bruker SMART CCD area-detector diffractometer	3144 independent reflections
Radiation source: fine-focus sealed tube	1786 reflections with *I* > 2σ(*I*)
graphite	*R*_int_ = 0.088
area detector ω scans	θ_max_ = 28.3°, θ_min_ = 2.2°
Absorption correction: multi-scan (*SADABS*; Bruker, 1998)	*h* = −12→11
*T*_min_ = 0.36, *T*_max_ = 0.58	*k* = −11→20
16026 measured reflections	*l* = −24→24

### Refinement

**Table:**

Refinement on *F*^2^	Primary atom site location: structure-invariant direct methods
Least-squares matrix: full	Secondary atom site location: difference Fourier map
*R*[*F*^2^ > 2σ(*F*^2^)] = 0.068	Hydrogen site location: inferred from neighbouring sites
*wR*(*F*^2^) = 0.222	H-atom parameters constrained
*S* = 1.01	*w* = 1/[σ^2^(*F*_o_^2^) + (0.1002*P*)^2^ + 6.7959*P*] where *P* = (*F*_o_^2^ + 2*F*_c_^2^)/3
3144 reflections	(Δ/σ)_max_ = 0.006
181 parameters	Δρ_max_ = 1.68 e Å^−^^3^
0 restraints	Δρ_min_ = −1.19 e Å^−^^3^

### Special details

**Table:**

Geometry. All e.s.d.'s (except the e.s.d. in the dihedral angle between two l.s. planes) are estimated using the full covariance matrix. The cell e.s.d.'s are taken into account individually in the estimation of e.s.d.'s in distances, angles and torsion angles; correlations between e.s.d.'s in cell parameters are only used when they are defined by crystal symmetry. An approximate (isotropic) treatment of cell e.s.d.'s is used for estimating e.s.d.'s involving l.s. planes.
Refinement. Refinement of *F*^2^ against ALL reflections. The weighted *R*-factor *wR* and goodness of fit *S* are based on *F*^2^, conventional *R*-factors *R* are based on *F*, with *F* set to zero for negative *F*^2^. The threshold expression of *F*^2^ > σ(*F*^2^) is used only for calculating *R*-factors(gt) *etc*. and is not relevant to the choice of reflections for refinement. *R*-factors based on *F*^2^ are statistically about twice as large as those based on *F*, and *R*- factors based on ALL data will be even larger.

### Fractional atomic coordinates and isotropic or equivalent isotropic displacement parameters (Å^2^)

**Table:**

	*x*	*y*	*z*	*U*_iso_*/*U*_eq_	
Ag1	0.60110 (7)	0.60171 (5)	0.51698 (3)	0.0574 (3)	
N1	0.5177 (6)	0.6250 (4)	0.6240 (3)	0.0412 (13)	
N2	0.4261 (7)	0.6635 (4)	0.7632 (3)	0.0506 (15)	
N3	0.8334 (7)	0.4221 (4)	0.9068 (3)	0.0454 (14)	
N4	0.7495 (6)	0.4621 (4)	0.7658 (3)	0.0408 (12)	
N5	0.5746 (7)	0.5612 (5)	0.8128 (3)	0.0473 (15)	
H5	0.5330	0.5710	0.8510	0.0541*	
C1	0.4145 (8)	0.6856 (5)	0.6372 (4)	0.0516 (18)	
H1	0.3721	0.7158	0.5988	0.062*	
C2	0.3696 (9)	0.7046 (6)	0.7056 (5)	0.059 (2)	
H2	0.2974	0.7474	0.7125	0.071*	
C3	0.5257 (7)	0.6012 (4)	0.7511 (4)	0.0402 (14)	
C4	0.5741 (7)	0.5822 (5)	0.6807 (4)	0.0398 (15)	
H4	0.6462	0.5394	0.6735	0.048*	
C5	0.6845 (7)	0.4989 (5)	0.8220 (3)	0.0396 (14)	
C6	0.7255 (7)	0.4780 (5)	0.8932 (3)	0.0442 (16)	
H6	0.6753	0.5042	0.9314	0.053*	
C7	0.9000 (7)	0.3833 (5)	0.8487 (4)	0.0442 (16)	
H7	0.9752	0.3424	0.8561	0.053*	
C8	0.8579 (8)	0.4036 (5)	0.7806 (4)	0.0477 (17)	
H8	0.9056	0.3760	0.7424	0.057*	
Cl1	0.1976 (2)	0.65255 (13)	0.43336 (10)	0.0500 (5)	
O1	0.1650 (12)	0.7083 (6)	0.4943 (4)	0.110 (3)	
O2	0.0655 (9)	0.6109 (6)	0.4123 (6)	0.115 (3)	
O3	0.2520 (7)	0.7041 (4)	0.3752 (3)	0.0724 (17)	
O4	0.3014 (9)	0.5867 (5)	0.4522 (5)	0.102 (3)	
O1W	0.3945 (6)	0.6282 (5)	0.9308 (3)	0.0656 (16)	
H1WB	0.3715	0.6732	0.9023	0.098*	
H1WA	0.4249	0.6335	0.9762	0.098*	

### Atomic displacement parameters (Å^2^)

**Table:**

	*U*^11^	*U*^22^	*U*^33^	*U*^12^	*U*^13^	*U*^23^
Ag1	0.0662 (5)	0.0630 (5)	0.0431 (4)	0.0062 (3)	0.0078 (3)	0.0003 (3)
N1	0.041 (3)	0.036 (3)	0.047 (3)	0.003 (2)	0.000 (2)	0.002 (2)
N2	0.052 (4)	0.046 (4)	0.054 (4)	0.015 (3)	0.004 (3)	−0.005 (3)
N3	0.048 (3)	0.045 (4)	0.043 (3)	−0.003 (3)	−0.004 (3)	0.003 (3)
N4	0.048 (3)	0.033 (3)	0.042 (3)	0.006 (2)	0.003 (2)	−0.001 (2)
N5	0.049 (3)	0.054 (4)	0.039 (3)	0.018 (3)	0.002 (3)	0.002 (3)
C1	0.050 (4)	0.047 (5)	0.058 (4)	0.005 (3)	−0.003 (3)	0.013 (4)
C2	0.064 (5)	0.045 (5)	0.069 (5)	0.021 (4)	0.007 (4)	0.006 (4)
C3	0.041 (4)	0.035 (4)	0.044 (3)	0.001 (3)	0.003 (3)	−0.003 (3)
C4	0.034 (3)	0.040 (4)	0.046 (4)	0.004 (3)	−0.001 (3)	−0.007 (3)
C5	0.038 (3)	0.039 (4)	0.042 (3)	0.001 (3)	0.004 (3)	−0.001 (3)
C6	0.046 (4)	0.050 (4)	0.037 (3)	0.001 (3)	0.004 (3)	−0.007 (3)
C7	0.042 (4)	0.038 (4)	0.052 (4)	0.001 (3)	−0.002 (3)	−0.005 (3)
C8	0.048 (4)	0.046 (4)	0.049 (4)	0.005 (3)	−0.005 (3)	−0.008 (3)
Cl1	0.0486 (9)	0.0443 (10)	0.0570 (10)	−0.0022 (8)	−0.0001 (8)	0.0074 (8)
O1	0.163 (8)	0.095 (7)	0.072 (4)	−0.002 (6)	0.036 (5)	−0.012 (4)
O2	0.074 (5)	0.111 (7)	0.161 (9)	−0.039 (5)	−0.023 (5)	0.010 (6)
O3	0.089 (4)	0.059 (4)	0.070 (4)	0.001 (3)	0.022 (3)	0.013 (3)
O4	0.095 (5)	0.080 (5)	0.131 (7)	0.034 (4)	0.014 (5)	0.053 (5)
O1W	0.069 (4)	0.070 (4)	0.058 (3)	0.016 (3)	−0.003 (3)	−0.003 (3)

### Geometric parameters (Å, °)

**Table:**

Ag1—N1	2.153 (6)	C1—H1	0.9300
Ag1—N3^i^	2.160 (6)	C2—H2	0.9300
N1—C1	1.334 (9)	C3—C4	1.407 (10)
N1—C4	1.337 (9)	C4—H4	0.9300
N2—C3	1.325 (9)	C5—C6	1.408 (9)
N2—C2	1.338 (10)	C6—H6	0.9300
N3—C6	1.317 (9)	C7—C8	1.355 (11)
N3—C7	1.368 (10)	C7—H7	0.9300
N3—Ag1^ii^	2.160 (6)	C8—H8	0.9300
N4—C5	1.321 (8)	Cl1—O2	1.406 (8)
N4—C8	1.350 (9)	Cl1—O4	1.415 (7)
N5—C3	1.370 (9)	Cl1—O3	1.420 (6)
N5—C5	1.382 (9)	Cl1—O1	1.444 (8)
N5—H5	0.8200	O1W—H1WB	0.8900
C1—C2	1.364 (12)	O1W—H1WA	0.8900
			
N1—Ag1—N3^i^	175.4 (2)	N1—C4—H4	119.6
C1—N1—C4	117.2 (6)	C3—C4—H4	119.6
C1—N1—Ag1	121.8 (5)	N4—C5—N5	120.8 (6)
C4—N1—Ag1	120.8 (4)	N4—C5—C6	121.9 (6)
C3—N2—C2	117.1 (7)	N5—C5—C6	117.3 (6)
C6—N3—C7	117.0 (6)	N3—C6—C5	121.2 (6)
C6—N3—Ag1^ii^	119.5 (5)	N3—C6—H6	119.4
C7—N3—Ag1^ii^	123.5 (5)	C5—C6—H6	119.4
C5—N4—C8	116.1 (6)	C8—C7—N3	120.8 (7)
C3—N5—C5	129.7 (6)	C8—C7—H7	119.6
C3—N5—H5	120.0	N3—C7—H7	119.6
C5—N5—H5	110.1	N4—C8—C7	122.9 (7)
N1—C1—C2	121.6 (7)	N4—C8—H8	118.5
N1—C1—H1	119.2	C7—C8—H8	118.5
C2—C1—H1	119.2	O2—Cl1—O4	108.2 (6)
N2—C2—C1	122.1 (7)	O2—Cl1—O3	109.3 (5)
N2—C2—H2	119.0	O4—Cl1—O3	110.3 (4)
C1—C2—H2	118.9	O2—Cl1—O1	108.0 (6)
N2—C3—N5	113.2 (6)	O4—Cl1—O1	110.9 (6)
N2—C3—C4	121.0 (7)	O3—Cl1—O1	110.0 (5)
N5—C3—C4	125.8 (6)	H1WB—O1W—H1WA	124.5
N1—C4—C3	120.8 (6)		
			
C4—N1—C1—C2	1.0 (11)	C8—N4—C5—N5	178.5 (7)
Ag1—N1—C1—C2	−175.2 (6)	C8—N4—C5—C6	−0.3 (10)
C3—N2—C2—C1	−1.8 (13)	C3—N5—C5—N4	−7.3 (12)
N1—C1—C2—N2	0.0 (13)	C3—N5—C5—C6	171.5 (7)
C2—N2—C3—N5	−178.4 (7)	C7—N3—C6—C5	−2.2 (10)
C2—N2—C3—C4	2.7 (11)	Ag1^ii^—N3—C6—C5	176.6 (5)
C5—N5—C3—N2	−174.7 (7)	N4—C5—C6—N3	1.7 (11)
C5—N5—C3—C4	4.1 (13)	N5—C5—C6—N3	−177.1 (7)
C1—N1—C4—C3	−0.1 (10)	C6—N3—C7—C8	1.4 (10)
Ag1—N1—C4—C3	176.2 (5)	Ag1^ii^—N3—C7—C8	−177.3 (5)
N2—C3—C4—N1	−1.8 (10)	C5—N4—C8—C7	−0.4 (11)
N5—C3—C4—N1	179.4 (7)	N3—C7—C8—N4	−0.1 (12)

Symmetry codes: (i) −*x*+3/2, −*y*+1, *z*−1/2; (ii) −*x*+3/2, −*y*+1, *z*+1/2.

### Hydrogen-bond geometry (Å, °)

**Table:**

*D*—H···*A*	*D*—H	H···*A*	*D*···*A*	*D*—H···*A*
N5—H5···O1Wi	0.82	2.12	2.911 (1)	162
O1W—H1WB···O3^iii^	0.89	2.21	3.036 (9)	154
O1W—H1WA···O2^iv^	0.89	2.45	3.306 (14)	161
O1W—H1WA···O1^iv^	0.89	2.51	3.063 (11)	121

Symmetry codes: i; (iii) *x*, −*y*+3/2, *z*+1/2; (iv) *x*+1/2, *y*, −*z*+3/2.
